# Overcoming analytical and preanalytical challenges associated with extragenital home collected STI specimens

**DOI:** 10.1128/jcm.00311-24

**Published:** 2024-06-05

**Authors:** B. E. Hockman, M. Qi, H. Rotblatt, L. Borenstein, R. A. Flynn, R. A. Muldrow, S. Rajagopalan, D. N. Greene

**Affiliations:** 1 LetsGetChecked Laboratories, Monrovia, California, USA; 2 Los Angeles County Department of Public Health Division of HIV and STD Programs, Los Angeles, California, USA; 3 Los Angeles County Department of Public Health Laboratory, Los Angeles, California, USA; 4 Los Angeles LGBT Center Clinic, Los Angeles, California, USA; 5 Laboratory Medicine and Pathology, University of Washington, Seattle, Washington, USA; Medical College of Wisconsin, Milwaukee, Wisconsin, USA

**Keywords:** extra-genital, self-collection, STI, home collection, chlamydia, gonorrhea, home screening, LGBT, anal, oropharyngeal

## Abstract

Home sample collection for sexually transmitted infection (STI) screening options can improve access to sexual healthcare across communities. For *Chlamydia trachomatis* (CT) and *Neisseria gonorrhoeae* (NG), genital infections have classically been the focus for remote collection options. However, infections may go undiagnosed if sampling is limited to urogenital sites because some individuals only participate in oral and/or anal intercourse. Here we evaluated samples for CT/NG detection after several pre-analytical collection challenges. A paired provider to self-collection validation was performed on rectal [*n* = 162; 22 + for CT and 9 + for NG by provider-collected (PC)] and throat (*N* = 158; 2 + for CT and 11 + for NG by provider-collected) swabs. The positive percent agreement for CT and NG ranged from 90.9% to 100%. The discrepancies were more often positive on self-collected (SC) (*n* = 9 SC+/PC−; *n* = 1 PC+/SC−; *n* = 1 PC+/SC Equiv.; *n* = 2 PC−/SC Equiv.). An empirical limit of detection (LoD) lower than the manufacturer’s claim (0.031 vs 2.5 IFU/mL for CT and 0.063 vs 124.8 CFU/ml for NG, respectively) was used to challenge additional variables. Common hand contaminants, including soap, hand sanitizer, lotion, and sunscreen were added to known positive (3× empirical LoD) or negative samples and did not influence detection. Samples at 2× and 10× the empirical LoD were challenged with extreme temperature cycling and extended room temperature storage. Detection was not affected by these conditions. These results indicate that remote self-collection is an appropriate method of sample acquisition for detecting extragenital CT/NG infections. Additionally, they provide a foundation towards meeting the regulatory standards for commercial testing of home collected extragenital samples.

**IMPORTANCE:**

There is a clinical need for expanded extragenital bacterial sexually transmitted infection (STI) testing options, but the current regulatory landscape limits the wide-spread promotion and adoption of such services. Improved access, particularly for the LGBTQ+ community, can be achieved by validating testing for specimens that are self-collected at a remote location and arrive at the laboratory via a postal carrier or other intermediary route. Here we provide valuable data showing that self-collected samples for anal and oropharyngeal STI testing are equally or increasingly sensitive compared with those collected by a provider. We systematically consider the effects of storage time, exposure to temperature extremes, and the addition of common toiletries on results.

## INTRODUCTION

*Chlamydia trachomatis* (CT) and *Neisseria gonorrhoeae* (NG) are the first and second most commonly reported bacterial infectious diseases in the United States, respectively (1). These sexually transmitted infections (STIs) are often asymptomatic across genders, delaying diagnosis and increasing the likelihood of complications and potentially irreversible health consequences (1–3). While CT and NG are traditionally recognized as genital infections, extragenital infections at rectal and oropharyngeal (referred to as throat from here on) sites are also common.

Extragenital STIs are usually identified in those engaging in receptive anal or oral intercourse but are also prevalent in those who do not report associated high-risk behaviors (1, 4–7). The highest prevalence is observed among men who have sex with men (MSM)(1, 4–7). Studies have also indicated increased rates of CT and NG infections, including extragenital infections, among transgender women similar to those observed in cisgender MSM (1, 4–7). However, extragenital infections are also common in cisgender women regardless of self-reported risk behaviors and cisgender men who report only having sex with cisgender women (MSW)(8, 9). According to a 2016 literature review, the prevalence of extragenital infections can vary considerably across studies and communities. For rectal and throat NG and/or CT infections, the median prevalence ranged from 1.7% to 8.9% among MSM and women, and 1.6% to 8.7% among MSW (1, 9).

The long-term consequences of untreated extragenital CT and NG infection are not well-understood, but they are known to contribute to onward transmission to those of all genders and sexual orientations (8). If left untreated, symptomatic and asymptomatic urogenital CT/NG vaginal infections can lead to pelvic inflammatory disease and irreversible infertility (10–12). Babies born from an infected parent may have serious health complications including a higher risk of inclusion conjunctivitis and pneumonia infections (13–15). CT and NG are also associated with HIV infection, and repeat infections at any site lead to a substantially higher risk of acquiring HIV, regardless of sex (1, 16–18).

It is estimated that approximately 70% of NG and CT infections may go undiagnosed if urogenital only testing is performed since the majority of rectal and throat infections are asymptomatic (1, 19–23).

The Centers for Disease Control and Prevention (CDC) suggests screening for extragenital infection based largely on reported sexual activity; however, extragenital infections are relatively common among those who both report and deny anal and oral sex (1, 8, 9). The prevalence of extragenital infections in men and women who deny high-risk sexual behavior may be due in part to avoidance of self-reporting caused by fear of scrutiny and discrimination. In addition, the gender nonbinary and transgender community often reports discrimination in healthcare settings including denial of services and socioeconomic barriers leading to avoidance of preventative care services and delayed treatment (1, 24–32). Therefore, screening and detection may increase with the availability of pseudo-anonymous testing options that do not require an in person visit to a healthcare facility. The term pseudo-anonymous is used because at-home sample collection does not require an in-person visit with a healthcare provider, offering a feeling of anonymity to the patient; however, patient identifying information is required for processing and reporting of results.

The CDC suggests that self-collected (SC) samples for the detection of urogenital (urine, vaginal swab) as well as extragenital (rectal swabs, throat swabs) infections are reasonable alternatives to provider-collected (PC) samples (1). The availability of home STI sample collection and screening has increased in recent years with the first at-home sample collection kit for CT and NG detection granted marketing authorization by the Food and Drug Administration (FDA) through the *de novo* pathway in 2023 (LetsGetChecked, Inc. Simple 2 Test) (33–35). However, SC testing options are largely limited to urine and vaginal swabs limiting the detection of extragenital infections, particularly in hard to reach populations that are unable or unwilling to visit a healthcare provider. The objective of this study was to: (i) demonstrate the effectiveness of within-clinic extragenital sample SC as a first step towards expansion to at home collection and (ii) elucidate the analytical and pre-analytical variables associated with at-home SC extragenital STI sample collection with the intention of improving inclusion and access to sexual healthcare.

## MATERIALS AND METHODS

### Study design, sample collection, and processing

Residual discard rectal and throat swabs, previously reported to be negative or positive for CT and/or NG by the Los Angeles County Department of Public Health (LACDPH, Downey, CA) or Kaiser Permanente Northern California (KPNC), were used for analytical method validation on the Hologic Panther CT/NG assay.

Clinical validation of SC rectal and throat swabs was performed using paired PC and SC rectal and throat samples collected at the Los Angeles LGBT Center Clinic (LA LGBT Center, Los Angeles, CA) from consenting individuals. The LA LGBT Center patient population has a high prevalence of STIs, and therefore every client visiting the sexual health program for HIV/STI counseling is tested for CT, NG, Syphilis, and HIV, regardless of whether their encounter is for routine testing, because of symptoms, or due to possible exposure. The standard of care for CT/NG is collection of rectal, throat, and urine specimens.

For rectal samples, the swab was inserted 1–2 inches (3–5 cm) past the anal margin and then rotated for 5–10 seconds. For throat samples, the swab was inserted into the mouth making contact with the tonsils on both sides then withdrawn without making contact with the cheeks or tongue. For both sample types, after collection, the single swab was placed into a Hologic Multitest Swab Specimen Collection container such that the swab tip was submerged in sample transport media (STM) so that the swab shaft could be broken at the score line. Participants collected samples using written instructions including diagrams and/or instructional videos prepared by the LA LGBT Center and LACDPH (Fig. S1 to S3) that were based on patient collection instructions for use created by Hologic. For all paired collections, the SC sample was obtained before the PC sample.

*In vitro* experiments were performed using live organisms with known titers purchased from Zeptometrix (Buffalo, NY) spiked into CT/NG negative rectal or throat swab matrix using a serial dilution method in Hologic Aptima Buffer (their standard sample transport media) to create desired concentrations. The product information of microorganism live titers provided by Zeptometrix is as follows. *C. trachomatis*, serovar D (2.04 × 10^9^ IFU/mL, lot #331047) was propagated in L929 host cells and frozen in sucrose-phosphate-glutamate (SPG) buffer. *N. gonorrhoeae* (1.14 × 10^8^ CFU/mL, lot #328175) was provided as a bacterial cell suspension frozen in brain–heart infusion broth with 15% glycerol.

All samples for clinical validation were collected under an IRB approved protocol issued to the LACDPH (LAC Public Health IRB, No. 2020–12-913) and then de-identified before sending to LetsGetChecked Labs for parallel analysis. All samples for *in vitro* experiments were collected under IRB approved protocols issued to LetsGetChecked Laboratories (Advarra IRB Pro00027040, IRB Organization Number: 0000635; IRB Registration Number: 00000971; Columbia, MD).

### Test method

CT/NG detection was performed using the Hologic Aptima Combo 2 Nucleic Acid Amplification Assay on the Panther system, which is FDA cleared for PC rectal and throat swabs.

### Validation of self-collected rectal and throat swabs

First, as an analytical verification of the instrumentation and sample matrix, known positive and negative rectal (CT, reference method: *n* = 24 positive/37 negative/0 equivocal; CT, comparator method: *n* = 23 positive/37 negative/1 equivocal; NG, reference and comparator methods: *n* = 21 positive/40 negative/0 equivocal) and throat swabs (reference and comparator methods: *n* = 11 positive/11 negative CT/0 equivocal CT; *n* = 12 positive/10 negative/0 equivocal NG) were used to evaluate if the specific matrix would interfere with testing.

Then, for validation of self-collected samples, paired provider-collected (PC) and self-collected (SC) rectal (*n* = 164) and throat (*n* = 159) swabs were compared for CT/NG detection at LetsGetChecked Laboratories. Samples that gave equivocal results on the Panther system were repeated using the same assay. If the repeat result was positive it was considered positive; if the repeat result was negative it was considered negative. If the repeat result remained equivocal or if the equivocal sample was quantity not sufficient for repeat, statistical analysis was performed with these results considered positive or negative for comparison. If any samples within a SC/PC pair provided a result of invalid, the sample pair was excluded from analysis (*n* = 2 rectal, *n* = 1 throat). Invalid results are caused by an array of sampling, reagent, or hardware issues and are provided with an error code on the report that indicates volume insufficiency, increased viscosity, inadequate reagent volumes, bubbles, or other issues. If the results from the PC and SC were discordant, we referred to the LACDPH results to adjudicate the discrepancy.

The PC and SC data sets for each microorganism and sampling site combination were statistically compared using the McNemar’s Chi squared (χ^2^) two-tailed *P*-value (with the continuity correction) and Cohen’s kappa score (κ). McNemar’s test provides an indication of a statistically significant difference between two data sets, and the continuity correction is recommended when the sum of the discordant results is small (<25) (36–39). When there are no discordant results the denominator of the equation to determine Chi squared becomes zero; in these cases, Chi squared is unable to be determined and is reported here as infinity (∞). Cohen’s kappa score is a measure of inter- and intra-rater reliability accounting for the possibility of agreement between two data sets occurring by chance (40–43).

### Deriving parameters for pre-analytical challenges

The LoD used was based on the FDA authorized Simple 2 Swab Home Collection Kit (Vaginal) that determined the empirical LoD for the Hologic Combo 2 Assay in vaginal swab matrix, as follows (33, 34). A preliminary screen to determine the vaginal swab CT/NG LoD was performed by testing five replicates at several concentrations of pathogen. Following, the LoD was challenged by testing 20 replicates at the lowest concentration(s) from which 100% detection was achieved during the preliminary screen. The LoD was defined as the lowest concentration at which ≥95% detection was achieved with 20 replicates and is henceforth referred to as the “empirical LoD” to differentiate from the LoD reported in the Hologic package insert. The empirical LoD was 0.031 IFU/mL and 0.063 CFU/mL for CT and NG, respectively, which is considerably lower than what is currently claimed for vaginal swabs in the Hologic package insert that is not cleared for remote collection (2.5 IFU/mL for CT and 124.8 CFU/mL for NG)(34). The assay components are identical, but the Simple 2 Test is authorized for at home sample collection as it provides instructions and consumables designed to facilitate at home collection. The differences between the LoDs indicated by the Simple 2 Test manufacturer (LetsGetChecked, Inc.) and the Combo 2 assay manufacturer (Hologic, Inc.) are a function of how the experiments were designed relative to the comparator and not due to analytical differences in reagents or instrumentation. A pathogen concentration of 2–10× the empirical LoD for vaginal swabs was used to challenge subsequent rectal and throat swab-based experiments.

#### Interference testing

Common hand contaminants (1% vol/vol) including soap, hand sanitizer, lotion, and sunscreen (three brands of each) were introduced to rectal and throat swab samples that were negative or low positive (at 3× empirical LoD) for CT or NG. Each hand contaminant was tested over three replicates in rectal and throat swab matrices to determine the potential for positive and negative interference, respectively. Tap water (1% vol/vol) was also included as a negative control.

#### Shipping stability

Self-collected throat and rectal swab samples were challenged using summer or winter simulation with temperature cycling designed to mimic extreme seasonal fluctuations. Samples were challenged for 56 hours cycling over temperatures spanning from 22 to 40°C (summer) and −10 to 18°C (winter) using a modified ISTA 7D 2007 shipping standard (guidelines recommended by the FDA for emergency use authorization submissions of home collection devices for molecular diagnostic detection of SARS-CoV-2). Extra-genital swabs (*n* = 20 throat and 20 rectal) were collected following Hologic’s instructions for use (IFU) into individual collection tubes and pooled. The pools were confirmed negative using the Hologic Combo 2 assay before aliquoting and spiking with CT or NG to 2× and 10× the empirical LoD with 10 µL (2×; *n* = 3/matrix/pathogen) or 50 µL (10×; *n* = 3/matrix/pathogen), respectively. The negative matrix without spiking (*n* = 3) was also challenged during temperature cycling experiments. Sample tubes were prepared containing the pooled matrix at the desired concentrations along with a Hologic multitest swab then placed into an incubator for temperature cycling.

#### Ambient stability

During the preparation of samples for both summer and winter shipping stability studies, a single additional sample for each pathogen/matrix/concentration/temperature challenge was prepared. These samples were screened prior to initiating the temperature cycling to ensure that contrived samples provided expected results prior to the stability challenges. The samples were then stored at room temperature and retested after 43 days to obtain ambient stability data.

## RESULTS

### Analytical validation of rectal and throat swabs

Analytical validation was completed to ensure that the sample matrix did not interfere with the assay or instrument performance. For CT, relative to the reference method, rectal swabs (*n* = 61; reference method: *n* = 24 positive/37 negative/0 equivocal; comparator method: *n* = 23 positive/37 negative/1 equivocal) demonstrated an overall agreement of 100% when equivocal results were considered positive (χ^2^=∞, *P* = 0.00, κ = 1.00) and 98.4% when equivocal results were considered negative (χ^2^ = 0.00, *P* = 1.00, κ = 0.965). Persistently equivocal CT results were observed in one rectal sample; this sample was reported as positive at the comparator lab. For NG, rectal swabs (*n* = 61; reference and comparator methods: *n* = 21 positive/40 negative/0 equivocal) demonstrated an overall agreement of 100% (χ^2^=∞, *P* = 0.00, κ = 1.00). Throat swabs (*n* = 22) demonstrated an overall agreement of 100% for both CT (reference and comparator methods: *n* = 11 positive/11 negative/0 equivocal; χ^2^=∞, *P* = 0.00, κ = 1.00) and NG (reference and comparator methods: *n* = 12 positive/10 negative/0 equivocal; χ^2^=∞, *P* = 0.00, κ = 1.00).

### Validation of self-collected rectal and throat swabs

#### CT detection in rectal swab samples

Self-collection validation was performed to ensure that CT/NG detection is not negatively affected by SC. For CT detection, relative to PC samples, rectal swabs (*n* = 162) demonstrated a positive agreement of 95.5% (*n* = 22 positive PC) with an overall agreement of 97.5% when equivocal results were considered positive (Table 1, χ^2^ = 0.25, *P* = 0.617, κ = 0.899). When equivocal results were considered negative, rectal swabs for CT detection, relative to PC, demonstrated a positive agreement of 90.9% (*n* = 22 positive PC) and an overall agreement of 97.5% (Table 2, χ^2^ = 0.25, *P* = 0.617, κ = 0.895).

**TABLE 1:** Qualitative accuracy of CT detection in paired rectal swabs (equivocal results considered positive)

Comparator method (self collected)	Reference method (provider collected)		
	CT positive	CT negative	Total
CT positive	21	3	24
CT negative	1	137	138
Total	22	140	162

**TABLE 2:** Qualitative accuracy of CT detection in paired rectal swabs (equivocal results considered negative)

Comparator method (self collected)	Reference method (provider collected)		
	CT positive	CT negative	Total
CT positive	20	2	22
CT negative	2	138	140
Total	22	140	162

There were five discordant pairs between PC (*n* = 22 positive/140 negative/0 equivocal) and SC (*n* = 22 positive/138 negative/2 equivocal). The first pair was CT positive by PC and CT negative by SC. When run at the LACDPH these samples were concordant negative. This suggests that the PC sample was at a very low bacterial load. The second and third discrepant pairs were CT negative by PC and CT positive by SC. Results at the LACDPH were consistent with these findings. This indicates that the SC sample was more sensitive than the PC sample. The fourth pair was CT negative by PC and CT equivocal by SC. SC sample volume was insufficient to retest at LetsGetChecked, and when evaluated at the LACDPH the results were identical (the PC swab was CT negative and the SC swab was CT equivocal). Similar to the second and third discrepant samples, this indicates that the SC sample was more sensitive than the PC sample. The last pair was CT positive by PC and CT equivocal by SC. When evaluated at LACDPH, the results were CT positive by PC and CT negative by SC. This indicates that the PC sample was more sensitive than the SC sample.

Invalid results were obtained in two SC rectal samples, both with RDFS and VVFS flags, and their PC pairs were CT negative. Upon testing at LACDPH, one of these samples provided invalid results and the other was CT negative; their PC pairs were both CT negative.

#### NG detection in rectal swab samples

For NG detection relative to PC samples, rectal swabs (*n* = 162) demonstrated a positive agreement of 100% (*n* = 9 positive PC) with an overall agreement of 98.1% (Table 3, χ^2^ = 1.33, *P* = 0.248, κ = 0.847).

**TABLE 3:** Qualitative accuracy of NG detection in paired rectal swabs

Comparator method (self collected)	Reference method (provider collected)		
	NG positive	NG negative	Total
NG positive	9	3	12
NG negative	0	150	150
Total	9	153	162

There were three discordant pairs between PC (*n* = 9 positive/153 negative/0 equivocal) and SC (*n* = 12 positive/150 negative/0 equivocal). All three discordant pairs were NG negative on PC and NG positive by SC. Results were consistent when evaluated at the LACDPH indicating that SC swabs were more sensitive than PC.

Invalid results were observed in two SC rectal samples, both with RDFS and VVFS flags, and their PC pairs were NG negative. Upon testing at LACDPH, one of these samples provided invalid results and the other was NG negative; their PC pairs were both NG negative.

#### CT detection in throat swab samples

For CT detection, relative to PC samples, throat swabs (*n* = 158) demonstrated a positive agreement of 100% (*n* = 2 positive PC) with an overall agreement of 100%. (Table 4, χ^2^=∞, *P* = 0.00, κ = 1.00). There were zero discordant pairs between PC (*n* = 2 positive/156 negative/0 equivocal) and SC (*n* = 2 positive/156 negative/0 equivocal) samples.

**TABLE 4:** Qualitative accuracy of CT detection in paired throat swabs

Comparator method (self collected)	Reference method (provider collected)		
	CT positive	CT negative	Total
CT positive	2	0	2
CT negative	0	156	156
Total	2	156	158

Invalid results were observed in one SC throat sample with RDFS and VVFS flags; its PC pair was CT negative. Upon testing at LACDPH, the sample was CT negative, and its PC pair was also CT negative.

#### NG detection in throat swab samples

For NG detection relative to PC samples, throat swabs (*n* = 158) demonstrated a positive agreement of 100% (*n* = 11 positive PC) with an overall agreement of 96.8% when equivocal results were considered positive (Table 5, χ^2^ = 3.20, *P* = 0.074, κ = 0.798). When equivocal results were considered negative, throat swabs for NG detection, relative to PC, demonstrated a positive agreement of 100% (*n* = 11 positive PC) and an overall agreement of 97.5% (Table 6, χ^2^ = 2.25, *P* = 0.134, κ = 0.833).

**TABLE 5:** Qualitative accuracy of NG detection in paired throat swabs (equivocal results considered positive)

Comparator method (self collected)	Reference method (provider collected)		
	NG positive	NG negative	Total
NG positive	11	5	16
NG negative	0	142	142
Total	11	147	158

**TABLE 6:** Qualitative accuracy of NG detection in paired throat swabs (equivocal results considered negative)

Comparator method (self collected)	Reference method (provider collected)		
	NG positive	NG negative	Total
NG positive	11	4	15
NG negative	0	143	143
Total	11	147	158

There were five discordant pairs between PC (*n* = 11 positive/*n* = 147 negative) and SC (*n* = 15 positive/*n* = 142 negative/*n* = 1 equivocal) samples; four discordant pairs were NG negative by PC and NG positive by SC; one discordant pair was NG negative by PC and NG equivocal by SC. Results for all five discordant pairs were consistent when evaluated at the LACDPH indicating that SC swabs were more sensitive than PC.

Invalid results were observed for one SC throat sample with RDFS and VVFS flags; its PC pair was NG negative. Upon testing at LACDPH the sample was NG negative, and its PC pair was also NG negative.

### Evaluation of analytical and pre-analytical variables associated with home sample collection

#### Interference from common hand contaminants

In the presence of all exogenous additives (1% vol/vol), throat and rectal swabs demonstrated overall positive and negative agreements of 100% at 3× empirical LoD specific to each organism (*n* = 3/sample type/contaminant/pathogen).

#### Shipping stability

After challenging with extreme temperature conditions, throat and rectal swabs demonstrated overall positive agreements of 100% at 2× and 10× empirical LoD specific to each organism (*n* = 3/sample type/concentration/temp profile). CT/NG negative throat and rectal samples demonstrated negative agreement of 100%.

#### Ambient stability

After storage at room temperature over 43 days, throat and rectal swabs had overall positive agreements of 100% at 2× and 10× empirical LoD specific to each organism (*n* = 2/sample type/concentration). CT/NG negative throat and rectal samples demonstrated negative agreement of 100%.

## DISCUSSION

There is an imminent need to increase the accessibility of comprehensive STI screening including detection of extragenital infections (1, 4–7). Federal agencies such as the CDC support patient collection of samples as an appropriate method for detection of NG and CT at both urogenital and extragenital sites (1). Even so, at-home sample collection options are often limited to detection of urogenital infections. In October 2023, the first commercial at home collection testing option was granted *de novo* marketing authorization by the FDA for CT/NG detection (LetsGetChecked, Inc. Simple 2 Test). While this is an important milestone in improving access to STI testing, the test is currently restricted to urine and vaginal swabs leaving a gap for detection of rectal and throat infections. There is a regulatory barrier to at-home collection STI screening for extragenital infections. To the best of our knowledge, large scale clinical studies required to support FDA authorization of extragenital genital sample self-collection within the clinic are absent among commercially available options of FDA cleared assays. As a first step toward supporting the quality requirements expected by regulatory bodies, we performed a comprehensive pre-analytical validation of self-collected rectal and throat swabs.

Here we demonstrate that self-collection is an appropriate method of sample acquisition for detecting extragenital CT/NG infections. SC samples were highly concordant to PC samples over a large number of samples at each collection site challenged. Percent positive and overall agreements were greater than 95% for all swab/organism combinations relative to PC samples. In fact, SC of extragenital samples may offer better sensitivity than PC without compromising the analytical prowess of the assay. Almost all discordant pairs were positive by SC and negative by PC (three SC rectal CT positive; three SC rectal NG positive; five SC throat NG positive), and only one discordant pair was negative by SC and positive by PC (one SC rectal CT positive). SC demonstrated equivalent sensitivity to PC among throat samples for CT detection and higher sensitivity than PC for all other scenarios. The infections that showed increased sensitivity for SC samples are all higher in prevalence than CT infections of the throat. Note that CT infections are not typical in throat tissue, making it difficult to obtain CT positive throat samples for validation. It is possible that a similar increase in sensitivity for the detection of CT in SC throat samples would have been observed if more positive samples had been challenged. Statistical analysis also confirmed excellent correlation between SC and PC data sets. Kappa (κ) scores were >0.81, indicating almost perfect agreement for all swab/organism data sets with one exception. For throat NG samples, when equivocal results were considered positive, κ was determined to be 0.798 suggesting substantial agreement (40–42). Similarly, McNemar’s test determined *P*-values indicative of a non-statistically significant difference between SC and PC for all swab/microorganism combinations whether equivocal results were considered positive or negative.

*In vitro* evaluation of analytical and pre-analytical variables associated with home collection of rectal and throat swab samples supported robust and sensitive detection of CT and NG. The concentrations of pathogens in the contrived samples used for these studies were derived from the empirical LoD determined for vaginal swabs described in the package insert for the FDA authorized Simple 2 Swab Home Collection Kit (Vaginal)(33, 34). At concentrations of 2×–10× the empirical LoD determined for vaginal swabs, the experiments described here were performed at concentrations well below the LoD claimed by the assay manufacturer for PC samples. No interference was detected in the presence of several common hand contaminants including soap, hand sanitizer, sunscreen, and lotion added at 1% of the total sample volume. Samples were stable for at least 56 hours during shipment with extreme hot and cold temperature conditions and at room temperature for at least 43 days.

There were several limitations to our study. (i) It cannot be ruled out that SC samples are more likely to be subject to contamination leading to false positivity as the cause of result discrepancies. This explanation is considered unlikely as other studies have also reported increased sensitivity with SC samples for STI testing (44). (ii) While SC of samples was not supervised by a clinician, the collection was in the clinic and not remotely based. Patients who knowingly make an error during in-clinic SC can be provided with a new collection kit immediately. In this study, we did not record if participants requested a second kit, had leaky specimens, or other manual errors. However, in the FDA submission for urine and vaginal swabs using the same device, participant comprehension of the critical information included in the IFU exceeded the acceptance criteria (≥ 95% success rate) as demonstrated through direct observation and sample rejection recorded at accessioning (45). Samples that are recieved by the laboratory for testing may be less likely to have collection errors if SC occurred in the clinic rather than at home where kit replacement is less convenient. Remote SC outside of a clinical environment could have a different outcome. (iii) No gender or other demographic information (age, race, ethnicity, education) was obtained with the collection validation samples preventing statistical analysis to discern if any demographic trends exist among discrepant samples that may suggest collection errors. (iv) Our interference studies included a range of contaminants at relatively high concentration, but it is possible that other brands or products could cause interference. (v) There may be other unexpected preanalytical variables that arise during sample home collection, which are difficult to predict, study, and detect. As home collection of samples becomes more common, unanticipated errors may be revealed that require additional studies. (vi) Studies supporting regulatory approval generally require more extensive testing than was feasible for this study. Clinical trials are often multi-centered studies including several hundred samples and an often enriched number of positive samples per condition relative to prevalence from both symptomatic and asymptomatic patients in the general public.

Nonetheless, these data support that home-collected STI specimens are a viable option for improving access to extragenital STI screening. For STI screening that effectively prevents ongoing transmission to be practical among all populations and communities, collection options allowing sample acquisition at a location and time that are discrete and convenient for the patient must be available. Home collection of samples offers a non-stigmatizing approach to sexual health without reducing the clinical performance of laboratory testing.

## SUPPLEMENTAL MATERIAL

Supplemental figures jcm.00311-24-s0001.docx Figures S1 to S3.

ASM does not own the copyrights to Supplemental Material that may be linked to, or accessed through, an article. The authors have granted ASM a non-exclusive, world-wide license to publish the Supplemental Material files. Please contact the corresponding author directly for reuse.

## References

[1] Workowski KA, Bachmann LH, Chan PA, Johnston CM, Muzny CA, Park I, Reno H, Zenilman JM, Bolan GA. 2021. Sexually transmitted infections treatment guidelines, 2021. Vol. 70. 10.15585/mmwr.rr7004a1 PMC8344968 34292926

[2] van der Helm JJ, Hoebe C, van Rooijen MS, Brouwers E, Fennema HSA, Thiesbrummel HFJ, Dukers-Muijrers N. 2009. High performance and acceptability of self-collected rectal swabs for diagnosis of Chlamydia trachomatis and Neisseria gonorrhoeae in men who have sex with men and women. Sex Transm Dis 36:493–497. doi:10.1097/OLQ.0b013e3181a44b8c 19617869

[3] Sexton ME, Baker JJ, Nakagawa K, Li Y, Perkins R, Slack RS, Baker DC, Jucha B, Arora S, Plankey MW. 2013. How reliable is self-testing for gonorrhea and chlamydia among men who have sex with men? J Fam Pract 62:70–78. 23405376 PMC12080727

[4] Allan-Blitz L-T, Konda KA, Calvo GM, Vargas SK, Leon SR, Segura ER, Caceres CF, Klausner JD. 2018. High incidence of extra-genital gonorrheal and chlamydial infections among high-risk men who have sex with men and transgender women in Peru. Int J STD AIDS 29:568–576. doi:10.1177/0956462417744098 29183269

[5] Hiransuthikul A, Janamnuaysook R, Sungsing T, Jantarapakde J, Trachunthong D, Mills S, Vannakit R, Phanuphak P, Phanuphak N. 2019. High burden of chlamydia and gonorrhoea in pharyngeal, rectal and urethral sites among Thai transgender women: implications for anatomical site selection for the screening of STI. Sex Transm Infect 95:534–539. doi:10.1136/sextrans-2018-053835 30982000

[6] Kojima N, Park H, Konda KA, Joseph Davey DL, Bristow CC, Brown B, Leon SR, Vargas SK, Calvo GM, Caceres CF, Klausner JD. 2017. The PICASSO cohort: baseline characteristics of a cohort of men who have sex with men and male-to-female transgender women at high risk for syphilis infection in Lima, Peru. BMC Infect Dis 17:255. doi:10.1186/s12879-017-2332-x 28399798 PMC5387233

[7] Pitasi MA, Kerani RP, Kohn R, Murphy RD, Pathela P, Schumacher CM, Tabidze I, Llata E. 2019. Chlamydia, gonorrhea, and human immunodeficiency virus infection among transgender women and transgender men attending clinics that provide sexually transmitted disease services in six US cities: results from the sexually transmitted disease surveillance network. Sex Transm Dis 46:112–117. doi:10.1097/OLQ.0000000000000917 30278030 PMC11155260

[8] Dewart CM, Bernstein KT, DeGroote NP, Romaguera R, Turner AN. 2018. Prevalence of rectal chlamydial and gonococcal infections: a systematic review. Sex Transm Dis 45:287–293. doi:10.1097/OLQ.0000000000000754 29465688 PMC6737334

[9] Chan PA, Robinette A, Montgomery M, Almonte A, Cu-Uvin S, Lonks JR, Chapin KC, Kojic EM, Hardy EJ. 2016. Extragenital infections caused by Chlamydia trachomatis and Neisseria gonorrhoeae: a review of the literature. Infect Dis Obstet Gynecol 2016:5758387. doi:10.1155/2016/5758387 27366021 PMC4913006

[10] STD facts - STD risk and oral sex. 2022. Available from: https://www.cdc.gov/std/healthcomm/stdfact-stdriskandoralsex.htm. Retrieved 10 Jan 2024.

[11] Detailed STD facts - Gonorrhea. 2023. Available from: https://www.cdc.gov/std/gonorrhea/stdfact-gonorrhea-detailed.htm. Retrieved 10 Jan 2024.

[12] Detailed STD facts - Chlamydia. 2023. Available from: https://www.cdc.gov/std/chlamydia/stdfact-chlamydia-detailed.htm. Retrieved 10 Jan 2024.

[13] Beem MO, Saxon EM. 1977. Respiratory-tract colonization and a distinctive pneumonia syndrome in infants infected with Chlamydia trachomatis. N Engl J Med 296:306–310. doi:10.1056/NEJM197702102960604 831128

[14] Frommell GT, Rothenberg R, Wang S, McIntosh K. 1979. Chlamydial infection of mothers and their infants. J Pediatr 95:28–32. doi:10.1016/s0022-3476(79)80077-3 480010

[15] Schachter J, Grossman M. 1981. Chlamydial infections. Annu Rev Med 32:45–61. doi:10.1146/annurev.me.32.020181.000401 7013681

[16] Pathela P, Braunstein SL, Blank S, Schillinger JA. 2013. HIV incidence among men with and those without sexually transmitted rectal infections: estimates from matching against an HIV case registry. Clin Infect Dis 57:1203–1209. doi:10.1093/cid/cit437 23800942

[17] Barbee LA, Khosropour CM, Dombrowksi JC, Golden MR. 2017. New human immunodeficiency virus diagnosis independently associated with rectal gonorrhea and chlamydia in men who have sex with men. Sex Transm Dis 44:385–389. doi:10.1097/OLQ.0000000000000614 28608786 PMC5481158

[18] Bernstein KT, Marcus JL, Nieri G, Philip SS, Klausner JD. 2010. Rectal gonorrhea and chlamydia reinfection is associated with increased risk of HIV seroconversion. J Acquir Immune Defic Syndr 53:537–543. doi:10.1097/QAI.0b013e3181c3ef29 19935075

[19] Patton ME, Kidd S, Llata E, Stenger M, Braxton J, Asbel L, Bernstein K, Gratzer B, Jespersen M, Kerani R, Mettenbrink C, Mohamed M, Pathela P, Schumacher C, Stirland A, Stover J, Tabidze I, Kirkcaldy RD, Weinstock H. 2014. Extragenital gonorrhea and chlamydia testing and infection among men who have sex with men--STD surveillance network. Clin Infect Dis 58:1564–1570. doi:10.1093/cid/ciu184 24647015 PMC4666527

[20] Kent CK, Chaw JK, Wong W, Liska S, Gibson S, Hubbard G, Klausner JD. 2005. Prevalence of rectal, urethral, and pharyngeal chlamydia and gonorrhea detected in 2 clinical settings among men who have sex with men: San Francisco, California, 2003. Clin Infect Dis 41:67–74. doi:10.1086/430704 15937765

[21] Koedijk FDH, van Bergen J, Dukers-Muijrers N, van Leeuwen AP, Hoebe C, van der Sande MAB, Dutch STI centres. 2012. The value of testing multiple anatomic sites for gonorrhoea and chlamydia in sexually transmitted infection centres in the Netherlands, 2006-2010. Int J STD AIDS 23:626–631. doi:10.1258/ijsa.2012.011378 23033514

[22] Barbee LA, Dombrowski JC, Kerani R, Golden MR. 2014. Effect of nucleic acid amplification testing on detection of extragenital gonorrhea and chlamydial infections in men who have sex with men sexually transmitted disease clinic patients. Sex Transm Dis 41:168–172. doi:10.1097/OLQ.0000000000000093 24521722

[23] Danby CS, Cosentino LA, Rabe LK, Priest CL, Damare KC, Macio IS, Meyn LA, Wiesenfeld HC, Hillier SL. 2016. Patterns of extragenital chlamydia and gonorrhea in women and men who have sex with men reporting a history of receptive anal intercourse. Sex Transm Dis 43:105–109. doi:10.1097/OLQ.0000000000000384 26766527 PMC4955797

[24] Poteat T, Reisner SL, Radix A. 2014. HIV epidemics among transgender women. Curr Opin HIV AIDS 9:168–173. doi:10.1097/COH.0000000000000030 24322537 PMC5947322

[25] White Hughto JM, Reisner SL, Pachankis JE. 2015. Transgender stigma and health: a critical review of stigma determinants, mechanisms, and interventions. Soc Sci Med 147:222–231. doi:10.1016/j.socscimed.2015.11.010 26599625 PMC4689648

[26] Radix AE, Lelutiu-Weinberger C, Gamarel KE. 2014. Satisfaction and healthcare utilization of transgender and gender non-conforming individuals in NYC: a community-based participatory study. LGBT Health 1:302–308. doi:10.1089/lgbt.2013.0042 26789858

[27] Rapues J, Wilson EC, Packer T, Colfax GN, Raymond HF. 2013. Correlates of HIV infection among transfemales, San Francisco, 2010: results from a respondent-driven sampling study. Am J Public Health 103:1485–1492. doi:10.2105/AJPH.2012.301109 23763398 PMC4007863

[28] Sevelius JM, Patouhas E, Keatley JG, Johnson MO. 2014. Barriers and facilitators to engagement and retention in care among transgender women living with human immunodeficiency virus. Ann Behav Med 47:5–16. doi:10.1007/s12160-013-9565-8 24317955 PMC3925767

[29] Sevelius JM. 2013. Gender affirmation: a framework for conceptualizing risk behavior among transgender women of color. Sex Roles 68:675–689. doi:10.1007/s11199-012-0216-5 23729971 PMC3667985

[30] Reisner SL, White Hughto JM, Pardee D, Sevelius J. 2016. Syndemics and gender affirmation: HIV sexual risk in female-to-male trans masculine adults reporting sexual contact with cisgender males. Int J STD AIDS 27:955–966. doi:10.1177/0956462415602418 26384946 PMC4798921

[31] Jaffee KD, Shires DA, Stroumsa D. 2016. Discrimination and delayed health care among transgender women and men: implications for improving medical education and health care delivery. Med Care 54:1010–1016. doi:10.1097/MLR.0000000000000583 27314263

[32] Glick JL, Theall KP, Andrinopoulos KM, Kendall C. 2018. The role of discrimination in care postponement among trans-feminine individuals in the U.S. national transgender discrimination survey. LGBT Health 5:171–179. doi:10.1089/lgbt.2017.0093 29589995 PMC6425922

[33] FDA Newsroom. 2023. FDA grants marketing authorization of first test for chlamydia and gonorrhea with at-home sample collection. FDA. Available from: https://www.fda.gov/news-events/press-announcements/fda-grants-marketing-authorization-first-test-chlamydia-and-gonorrhea-home-sample-collection

[34] Device classification under section 513(f)(2)(De Novo). Accessed 1 February 2024. https://www.accessdata.fda.gov/scripts/cdrh/cfdocs/cfpmn/denovo.cfm?id=DEN200070.

[35] Kersh EN, Shukla M, Raphael BH, Habel M, Park I. 2021. At-home specimen self-collection and self-testing for sexually transmitted infection screening demand accelerated by the COVID-19 pandemic: a review of laboratory implementation issues. J Clin Microbiol 59:e0264620. doi:10.1128/JCM.02646-20 34076475 PMC8525576

[36] McNemar Q. 1947. Note on the sampling error of the difference between correlated proportions or percentages. Psychometrika 12:153–157. doi:10.1007/BF02295996 20254758

[37] McNemar’s chi-square test. Available from: https://www2.ccrb.cuhk.edu.hk/stat/confidence%20interval/McNemar%20Test.htm#Formula. Retrieved 1818 AprApril 2024. Accessed , 1818 AprApril 2024

[38] Edwards AL. 1948. Note on the correction for continuity in testing the significance of the difference between correlated proportions. Psychometrika 13:185–187. doi:10.1007/BF02289261 18885738

[39] Results of McNemar’s test for a case-control study. Available from: https://www.graphpad.com/quickcalcs/mcNemar2/. Retrieved 2222 AprApril 2024. Accessed , 2222 AprApril 2024

[40] McHugh ML. 2012. Interrater reliability: the kappa statistic. Biochem Med (Zagreb) 22:276–282. 23092060 PMC3900052

[41] Cohen’s kappa free calculator – IDoStatistics. Available from: https://idostatistics.com/cohen-kappa-free-calculator/#risultati. Retrieved 1818 AprApril 2024. Accessed , 1818 AprApril 2024

[42] Quantify agreement with kappa. Available from: https://www.graphpad.com/quickcalcs/kappa2/. Retrieved 2222 AprApril 2024. Accessed , 2222 AprApril 2024

[43] Landis JR, Koch GG. 1977. The measurement of observer agreement for categorical data. Biometrics 33:159–174. doi:10.2307/2529310 843571

[44] Nyatsanza F, Trivedy A, Brook G. 2016. The effect of introducing routine self-taken extra-genital swabs in a genitourinary medicine clinic cohort: a before and after study. Int J STD AIDS 27:1330–1333. doi:10.1177/0956462415621833 26672002

[45] Simple 2 package insert. Letsgetchecked. Available from: https://www.letsgetchecked.com/simple2packageinsert/. Retrieved 2929 AprApril 2024. Accessed , 2929 AprApril 2024

