# Supplementary material for: Overcoming analytical and preanalytical challenges associated with extragenital home collected STI specimens
Source: J Clin Microbiol. 2024 Jun 5;62(7):e00311-24. doi: 10.1128/jcm.00311-24 (PMC11250113; doi:10.1128/jcm.00311-24)
Supplement: Supplemental figures — Figures S1 to S3. [file jcm.00311-24-s0001.docx]

**SUPPLEMENTAL MATERIAL**

**Supplemental Figure 1:** LA LGBT Center Clinic Instructions for self-collection of rectal swab sample

**Supplemental Figure 2:** LA LGBT Center Clinic Instructions for self-collection of throat swab sample


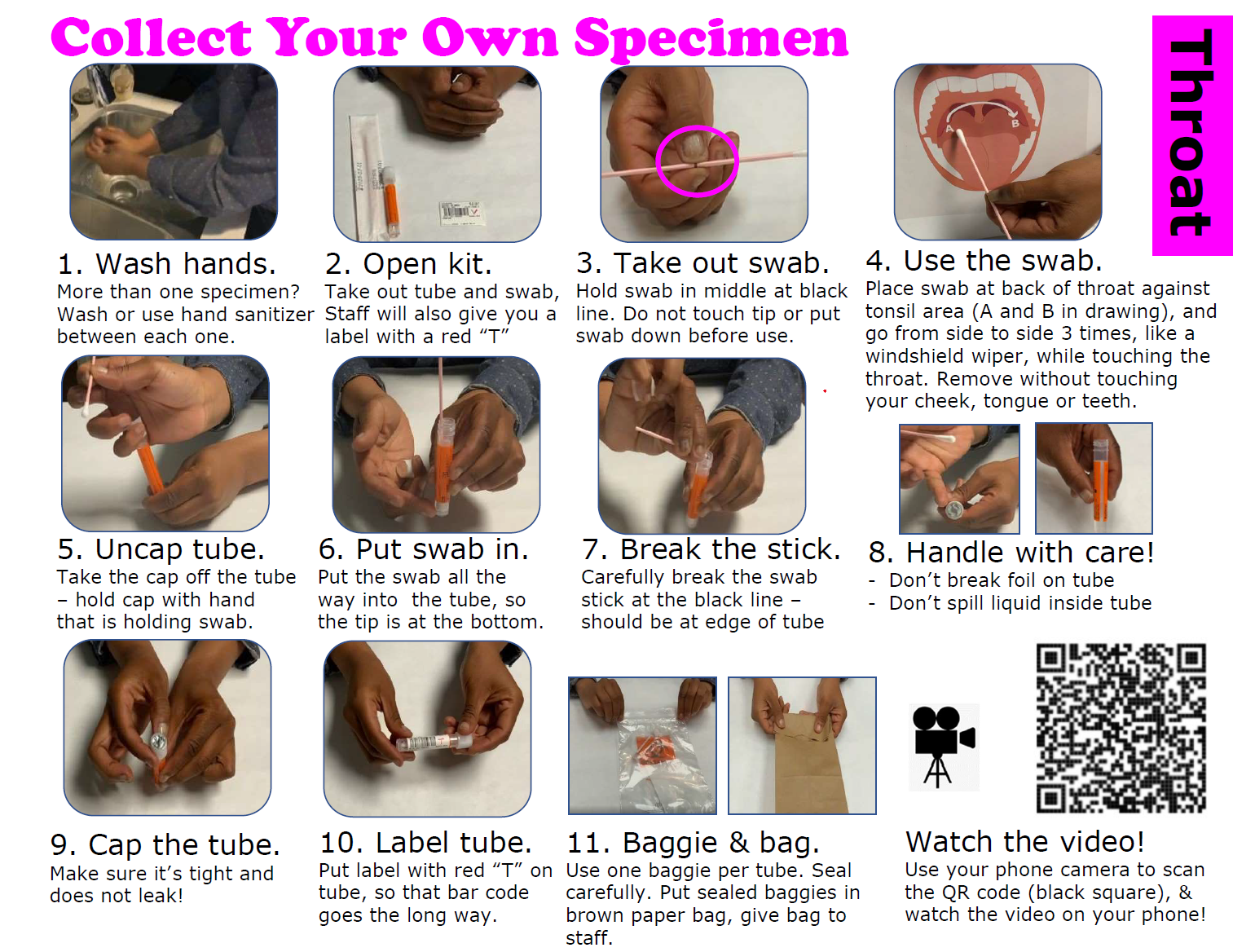


**Supplemental Figure 3:** LA LGBT Center Clinic Self-Collection Instructions

**
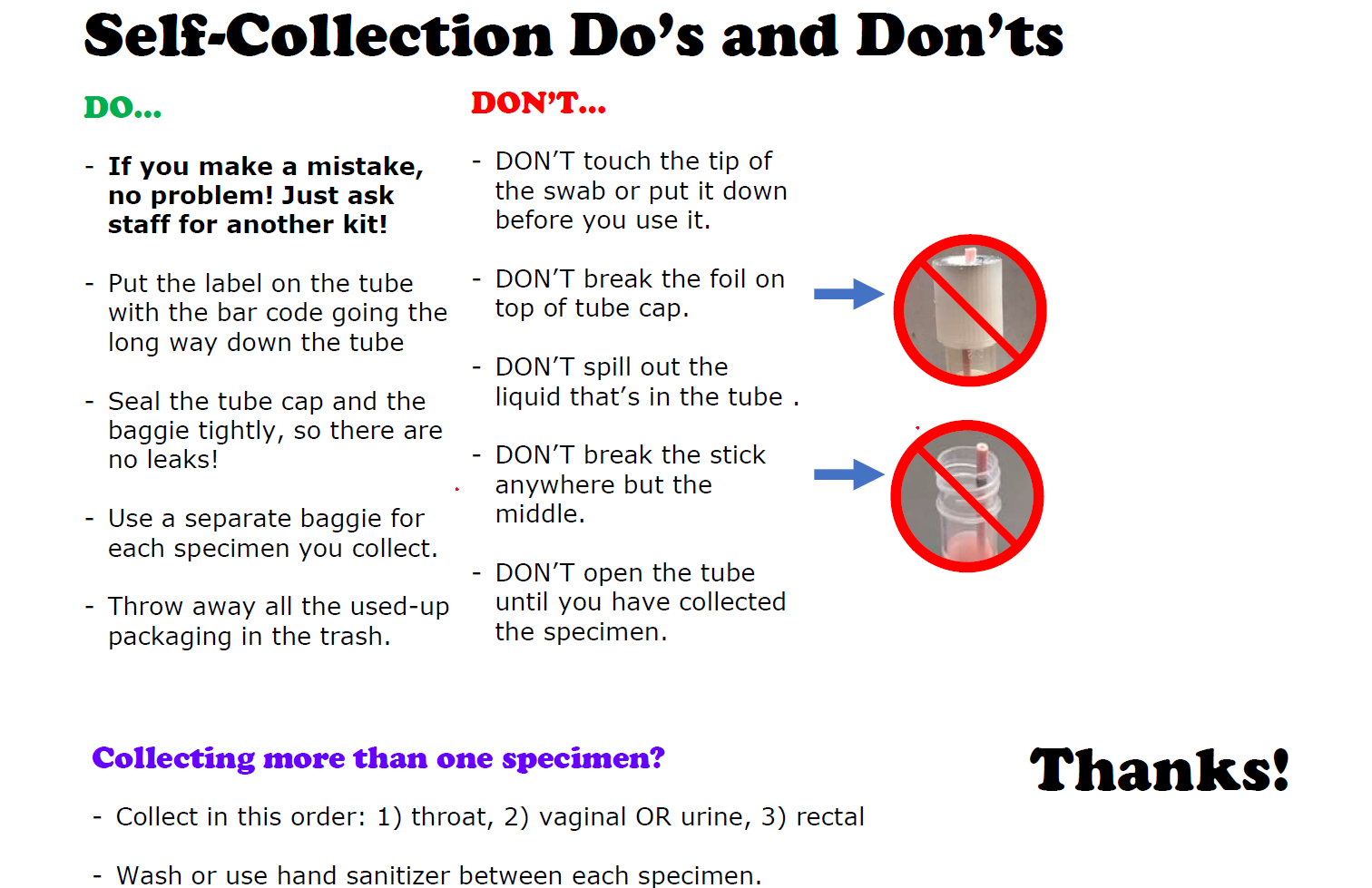
**
